# Metabolism of 2-oxo-clopidogrel to the clopidogrel active H4 metabolite using human liver microsomes and its inhibitory effect on ADP-induced human platelet aggregation *ex vivo*: a confirmatory, placebo-controlled, parallel-group study

**DOI:** 10.3389/fcvm.2026.1842832

**Published:** 2026-08-24

**Authors:** Christian Schulz, Steffen Braune, Christof Mrowietz, Jan-Heiner Küpper, Friedrich Jung

**Affiliations:** 1 Fraunhofer Institute for Cell Therapy and Immunology, Branch Bioanalytics and Bioprocesses (IZI-BB), Site Lausitz (IZI-BB-L), Fraunhofer-Gesellschaft zur Förderung der Angewandten Forschung e. V., Senftenberg, Germany; 2 Institute of Biotechnology, Department of Molecular Cell Biology, Brandenburg University of Technology Cottbus-Senftenberg, Senftenberg, Germany; 3 Faculty of Health Sciences Brandenburg, Brandenburg University of Technology Cottbus-Senftenberg, Senftenberg, Germany

**Keywords:** 2-oxo-clopidogrel, ADP-induced platelet aggregation, clopidogrel, H4 metabolite, human liver microsomes

## Abstract

Clopidogrel is a drug that is commonly used to prevent occlusive events after coronary artery interventions. However, the efficacy of clopidogrel varies depending on the CYP2C19 polymorphism, as it is not clopidogrel itself but the H4 metabolite that is responsible for the pharmacological effect. Carriers of the CYP2C19 loss-of-function allele genotype – which can affect up to 50% of patients - exhibited a higher likelihood of experiencing cardiovascular events. Administering the H4 metabolite directly avoids this problem. In the first step, the H4 metabolite was successfully produced *in vitro* from 2-oxo-clopidogrel using human liver microsomes. Thereafter, human platelet-rich plasma was supplemented with the H4 metabolite and then stimulated with adenosindiphosphate (ADP, 20 µM). The present confirmatory, placebo-controlled, parallel-group *ex vivo* study showed that the H4 metabolite of clopidogrel metabolized by human liver microsomes significantly and relevantly inhibited ADP-induced platelet aggregation. The study thus showed that the H4 metabolite of clopidogrel produced by human liver microsomes completely overcomes the limitations of clopidogrel, such as high inter-individual variability due to genetic polymorphisms of CYP2C19, a delayed onset of action and inadequate efficacy.

## 1 Introduction

The use of clopidogrel (CLP) in combination with aspirin is standard in the treatment of patients with acute coronary syndromes and/or stent implantation. CLP is a prodrug that requires metabolic activation *in vivo* to effectively decrease cardiovascular complications of atherosclerosis (1–4). The active metabolite of CLP inhibits the adenosine diphosphate (ADP) binding receptor P2Y_12_ on the platelet surface and thus prevents platelet aggregation and the formation of thrombi (5, 6). Clinical studies have shown that post-interventional hyperactive platelets are a risk factor for thromboembolic complications following cardiovascular surgery (7–10). Furthermore, findings from the large-scale CAPRIE clinical trial showed that CLP provided greater protection than aspirin against vascular ischemic events – including myocardial infarction, stroke, and vascular death – in patients with a history of symptomatic atherosclerotic disease (11).

CLP develops its intrinsic effect after a two-stage enzymatic activation by oxidation of the thiophene ring mainly by cytochrome-P450 (CYP) monooxygenases (CYP2C19, CYP1A2, CYP2B6) (12). First, a thiolactone (2-oxo-clopidogrel, 2-oxo CLP) is formed. CYPs convert this into the active thiol metabolite (the so-called H4 metabolite) (12). Moreover, *in vitro* studies have shown that the metabolic activation of CLP leads to a mixture of four stereoisomers (H1-H4). Plasma samples from patients receiving CLP showed that H3 and H4 are formed *in vivo*, with only H4 being active (13, 14). H4 selectively and irreversibly inhibits ADP-induced platelet aggregation by directly blocking the binding of ADP to the P2Y_12_ receptor on the platelet surface (15).

Genetic polymorphisms of drug-metabolizing enzymes and differences in the prevalence of variant alleles associated with altered enzyme function in different populations are the causes of intrinsic pharmacokinetic variability. The formation of the active H4 metabolite of CLP is related to CYP2C19 activity. 20%–40% of patients are categorized as ‘non’ or ‘low’ responders to CLP due to genetic polymorphisms (16), and clinical data in patients with acute ischemic stroke show that CYP2C19*2/*3 loss-of-function alleles are associated with lack of response to CLP (17). Beyond this association, a randomized controlled trial in patients with ischemic stroke or transient ischemic attack (TIA) demonstrated that personalized antiplatelet therapy guided by *CYP2C19* genotyping and clinical characteristics significantly reduced recurrent stroke and composite vascular events, and decreased disability without increasing major bleeding compared with standard treatment (18). CYP2C19*2 is the most common genotype of low responders in up to 50% of Asians and up to 15% of Caucasians (19–22), and has also been shown to be strongly associated with lack of response to CLP in acute ischemic stroke patients (17). Individuals with this genotype cannot achieve sufficient platelet inhibition when taking CLP. This is known as ‘clopidogrel resistance’ (8) and carries a higher risk of adverse cardiovascular events, such as stent thrombosis, myocardial infarction or stroke (7–10).

Since direct administration of the H4 metabolite is independent of genetic polymorphisms that may prevent the individual metabolization of CLP, it may represent a more effective treatment strategy. The application of the active metabolite could further reduce disadvantages that are associated with the inefficient biotransformation of the prodrug clopidogrel into H4 in the human liver. The metabolic capacity of the liver in cardiological patients is usually already highly burdened, as a significant number of these cardiovascular medications are subject to the hepatic metabolism (e.g., beta-blockers, calcium antagonists, antiarrhythmic drugs). The concomitant ingestion of multiple substances has been demonstrated to enhance the probability of interactions among them, thereby exerting a potentiated effect on the hepatic burden.

However, the administration of various pharmaceuticals is imperative for the management of cardiovascular diseases, particularly in elderly patients (23). In a significant number of cases, this results in gastrointestinal disorders, necessitating the administration of proton pump inhibitor medications to patients alongside their cardiovascular therapeutics (24).

Direct administration of the H4 metabolite via intravenous injection could reduce the burden on the liver and gastrointestinal tract of cardiovascular patients while enabling highly effective treatment of hyperreactive platelets (25–27). This strategy can reduce the formation of inactive CLP metabolites in the human liver, as it bypasses carboxylation by esterases for example. This reaction, which is catalyzed by the liver enzyme CES-1, inactivates approximately 85% of the administered CLP, thereby significantly reducing the amount of substrate available for H4 formation (28).

The present confirmatory study tested the hypothesis that the active H4 metabolite of clopidogrel - produced *in vitro* by human liver microsomes (HLM) - can significantly inhibit ADP-induced platelet aggregation.

## 2 Material and methods

### 2.1 Study design

The influence of the HLM-derived active H4 metabolite of CLP on ADP-induced platelet aggregation of human platelets was investigated in a confirmatory, placebo-controlled parallel group study *ex vivo*. The study was performed on blood samples from apparently healthy volunteers. As a placebo, a vehicle solution composed of assay buffer + all additives of the HLM-metabolization – except the substrate for the H4 metabolite synthesis – was used. The vehicle was shown not to influence platelet aggregation (see Table 2).

Before performing the confirmatory study five pre-studies were performed to generate required data.

All human studies were approved by the appropriate ethics committee of the Brandenburg Technical University Cottbus - Senftenberg (file number: EK2024-18) and were therefore conducted in accordance with the ethical standards set out in the 1964 Declaration of Helsinki and its subsequent amendments. All individuals gave their informed consent prior to participating in the study.

### 2.2 Generation of active clopidogrel metabolite H4 by human liver microsomes

In accordance with an study of Pereillo et al., we also found in a first study (data not shown) that synthesis of the active metabolite H4 directly from clopidogrel using HLM (pool of 50 donors, Thermo Fisher Scientific Inc., Waltham, MA, USA, HMMCPL, Lot: PL050J) resulted in very low yields and no platelet response could be achieved (29). Therefore, metabolization was performed using 2-oxo-clopidogrel (2-oxo CLP, Merck KGaA, Darmstadt, Germany) as substrate in the subsequent study. Metabolization was performed with 200 µL preparations in 2 mL amber glass vials (Agilent Technologies Inc., Santa Clara, CA, USA). 0.5 mM 2-oxo CLP (50 mM stock dissolved in DMSO) was metabolized with 200 µg HLM in potassium phosphate (KPO_4_) buffer (0.1 mM, pH 7.4) supplemented with 5 mM GSH (Merck KGaA) and twice the amount of NADPH regeneration system (Promega, Madison, WI, USA) suggested by the manufacturer at 37 °C for 90 min. After metabolization, samples were centrifuged at 16,000 × g for 15 min and 4 °C to stop the reaction and remove HLM from the solution. All HLM metabolization reactions to generate the H4-containing supernatants were performed freshly on the day of the platelet function experiments. Supernatants were either used immediately for *ex vivo* platelet aggregation testing or stored on ice at 4 °C for a maximum of 4 h before use. The supernatants obtained were used for platelet function studies, with comparable samples without addition of 2-oxo CLP serving as reference. To ensure a standardized and reproducible generation of H4, the same batch of commercial HLM (Lot: PL050J) was used throughout the study. Upon receipt, HLM were thawed once, aliquoted, and stored at −80 °C. For each metabolization reaction, fresh aliquots from this stock were used. All other critical reagents (2-oxo CLP, GSH, and NADPH regeneration system) were freshly prepared from solid or stock solutions and used as single-use aliquots to minimize variability between preparations, while all metabolization conditions (HLM protein amount, substrate concentration, buffer composition, incubation time and temperature) were kept constant for all reactions. In order to exclude an influence of the substrate remaining after the metabolic reaction on platelet aggregation, platelet rich plasma (PRP) was treated with 250 µM 2-oxo CLP and ADP-induced platelet aggregation was measured in comparison with untreated PRP. Adding 2-oxo CLP to PRP did not influence the ADP-induced platelet aggregation measured after 5 min incubation (*p* > 0.05, *n* = 7):

$$\begin{array}{rl} & TA_{\max},\;\mathrm{ADP}-\mathrm{induced}\;\mathrm{platelet}\;\mathrm{aggregation}\;\mathrm{with}\;\mathrm{vehicle}:\\ & \;\;\;\;\;\;76.5\;\pm \;16.1\; \end{array}\text{\%}$$

$$\begin{array}{rl} & TA_{\max},\;\mathrm{ADP}-\mathrm{induced}\;\mathrm{platelet}\;\mathrm{aggregation}\;\mathrm{with}\;\mathrm{vehicle}\;\\ & +\;2-oxo-clopidogrel:\;\;\;\;\;\;\;\;\;\;\;77.3\;\pm \;10.4\;\text{\%} \end{array}$$

For HPLC-based metabolite profiling and quantification, supernatants obtained after metabolization were immediately derivatized for 10 min at ambient temperature with 4 mM 2-bromo-3′-methoxyacetophenone (MPB; 400 mM stock dissolved in acetonitrile, Merck KGaA) to stabilize thiol-containing CLP metabolites, including H4, and to prevent their degradation during subsequent sample handling and analysis. Subsequently, the samples were mixed with an equal volume of ice-cold acetonitrile, and spiked with 100 µM phenacetin (Merck KGaA) as internal standard. After addition, the samples were vortexed, incubated on ice for 5 min and centrifuged with 16,000 x g at 4 °C for 15 min. Supernatants obtained were analyzed by HPLC. The setup consisted of a SCL-40 (SHIMADZU, Kyoto, Japan) with a ZORBAX SB-C18 column (Agilent Technologies Inc.). For metabolite separation, 20 µL sample was separated in a gradient of KH_2_PO_4_ buffer (10 mM, pH 3, mobile phase A) and acetonitrile (mobile phase B) at a flow rate of 1 mL/min and 50 °C column temperature. Metabolites were detected based on elution time using DAD (SPD-M40) at 220 nm. The gradient was: 0–3 min: 30% B, 20.5 min: 55% B, 22.5 min: 55% B, 23 min: 95% B, 26 min: 95% B, 26.5 min: 20% B, 29 min: 20% B and 30 min: 30% B. Quantification of detected metabolites was performed using standard solutions of commercially available references of MPB stabilized H3/H4 derivatives cis-clopidogrel MP (cis-CLP MP; pair of enantiomers) from LGC Standards GmbH (Wesel, Germany) and 2-oxo CLP from Merck KGaA. Identification of the CLP-derived peaks was based on matching retention times with those of the reference standards: 2-oxo-CLP appeared as a diastereomeric double peak at 19.4 and 19.9 min, H3 eluted at 22.1 min, and H4 at 22.7 min. For quantification, calibration curves were generated from the cis-clopidogrel MP (for H3/H4) and 2-oxo-CLP standards, and phenacetin served as an internal standard to monitor injection and system performance. Although a formal regulatory bioanalytical validation was not performed, the method showed sufficient specificity and reproducibility for the purpose of this *ex vivo* platelet function study. Stock solutions were dissolved in DMSO and further diluted in KPO_4_ buffer. HPLC analytics were performed with *n* = 6 independent HLM metabolizations. These independent metabolization reactions yielded highly consistent H3 and H4 concentrations with low standard deviations and stable peak areas of the internal standard phenacetin, indicating robust and reproducible microsomal activity and conversion efficiency across all preparations.

### 2.3 Study population

According to the Nordkem-workshop criteria, blood was collected from apparently healthy subjects, who had not received antiplatelet or other medications for at least 10 days (30, 31). Blood tests were performed to exclude subjects with early inflammatory processes and abnormalities in platelet count or function. ADP-induced platelet aggregation (citrated blood samples) and hemogram values, including of red- and white-blood cell counts, hemoglobin and hematocrit, were tested (EDTA anticoagulated whole blood, Sysmex XS-800i, Norderstedt, Germany). In addition, C-reactive protein levels were tested from EDTA anticoagulated whole blood utilizing a semi-quantitative quick test (Hangzhou Realy Tech co. Ltd., Hangzhou City, China).

All parameters were checked to be within the reference ranges; otherwise, the donor was excluded from the study. According to these criteria *n* = 8 participants were included in the confirmatory, placebo-controlled study. Four males and four females were included. The mean age was 38.8 ± 16.2 years with a body weight of 78.4 ± 18.4 kg and a height of 174.8 ± 4.5 cm and a body mass index of 25.6 ± 5.7. Mean systolic blood pressure was 133.2 ± 18.9 mmHg, diastolic 82.9 ± 12.3 mmHg and the heart rate 70.5 ± 20.6 beats/min.

### 2.4 Preparation of blood specimens

Following a standardized atraumatic protocol, blood was drawn from the cubital vein and collected in S-Monovettes® (Sarstedt, Nümbrecht, Germany), containing sodium citrate as an anticoagulant in a final concentration of 0.106 M (32). To homogenize the anticoagulant and the blood, the tubes were gently agitated immediately after blood collection and samples were discarded if there was any evidence of clotting. In accordance with the British Society of Haematology (33), donors were only included in the study if the platelet count was within the range of 150–600 × 10^9^ L^–1^. Platelet-rich plasma (PRP) and platelet-poor plasma (PPP) were obtained by centrifugation of citrated whole blood at 140 ×  g for 20 min, or at 1,500 ×  g for 20 min, respectively. PRP and PPP were used to prepare plasma containing 250 ×  10^3^ platelets/µL. This thrombocrit was used for all preliminary tests and for the confirmatory study.

### 2.5 Closure time (PFA100)

The PFA-100 (Siemens Healthcare, Erlangen, Germany) was utilized to assess the platelet function in sodium citrated whole blood (31) assessing the closure time in a capillary. Cartridges coated with collagen/epinephrine as well as with collagen + ADP were used to measure the closure times after exposition of the blood to the coating.

### 2.6 Platelet aggregation *in vitro*

Platelet aggregation was determined by the Born turbidimetric method (34) in a four-channel light transmission aggregometer (APACT-4004; Haemochrom Diagnostica, Essen, Germany). PRP was preincubated with the active H4-metabolite of clopidogrel for 5 min before stimulation of aggregation by addition of ADP (20 μM final, Sigma Aldrich, St. Louis, Missouri, USA) was performed. Aggregation index curves describing the changes in light transmission intensity were recorded for 10 min. The maximum extent of aggregation expressed as a percentage (TA_max_ [%]) and the slope of the aggregation graph's increase as STA [degree/min] were obtained in each single measurement for each donor. To minimize the effects of storage time on blood platelets and components (e.g., cell lesion and proteolytic protein degradation) and to ensure a proper platelet function, all platelet aggregation experiments were completed within 4 h after blood collection (35–37). Incubation of PRP with the H4-containing supernatants and subsequent ADP stimulation were carried out at 37 °C, and aggregation curves were recorded over a 10 min measurement period, thereby also limiting the exposure time of H4 under assay conditions.

### 2.7 Statistical analysis

The sample size estimation required a minimum sample of *n* = 8 (38). All data are expressed as mean ± standard deviation (SD). Data displayed Gaussian distribution. Intergroup differences were calculated using paired two-tailed Student´s t-test. Confirmatory parameter of the study is the maximum of the aggregation curve (TA_max_) after adding ADP to the PRP preincubated with the H4 metabolite for an incubation period of 6 min. The significance threshold was set at *p* ≤ 0.05. Statistical analyses were performed using GraphPad PRISM v8 (GraphPad Software®).

## 3 Results

The active metabolite of CLP, called H4, is synthesized in the liver via CYP450 enzymes with 2-oxo CLP as an intermediate. By contrast, H3 which is a diastereomer of H4 is not active on thrombocytes. We found that the formation of downstream metabolites in HLM is more efficient when 2-oxo CLP is provided as a substrate instead of the prodrug CLP. Following preliminary studies were carried out prior to the start of this confirmatory study: The concentrations of H3 and H4 metabolites – metabolized from 2-oxo CLP – as well as the amount of the remaining substrate were quantified. It was ensured that platelet function was within the reference range in all participants. It was tested whether the medium used without the H4 (vehicle solution) metabolite influenced platelet function. The influence of 2-oxo CLP on the platelet function was analyzed and the effective dose of the freshly produced H4 metabolite was determined. Taking this data into account, the confirmatory, placebo-controlled, parallel group study of the efficacy of the H4 metabolite on ADP-induced platelet aggregation was performed.

### 3.1 Concentration of the H4 metabolite

**Figure 1:**
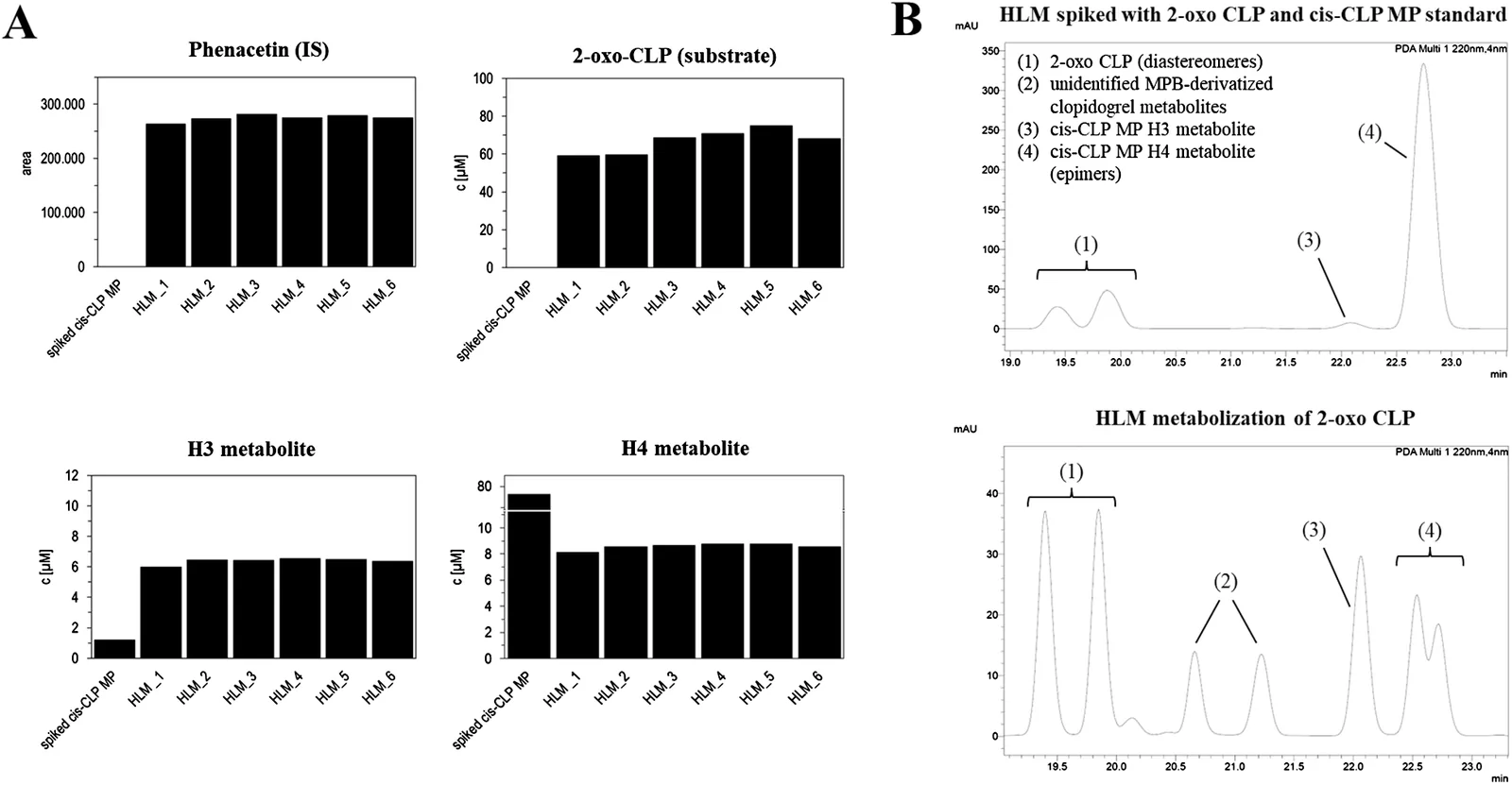
**(A)** Quantification of the clopidogrel metabolites H3 and H4 and the remaining 2-oxo-clopidogrel substrate in six independent human liver microsome (HLM) metabolization samples (HLM_1 - HLM_6). A vehicle sample spiked with 50 µM of the commercial cis-CLP MP standard served as reference for the identification and quantification of H3/H4 after MPB derivatization. All HLM samples were additionally spiked with phenacetin as internal standard. **(B)** Representative HPLC chromatograms. Upper chromatogram: HLM sample spiked with 50 µM 2-oxo CLP and cis-CLP MP. Lower chromatogram: Representative chromatogram of an HLM metabolization sample after 90 min incubation with 2-oxo CLP after MPB derivatization, showing (1) the diastereomeric double peak of residual 2-oxo CLP (∼19.4 and 19.9 min), (2) two additional, as yet unidentified CLP-related peaks (∼20.7 and 21.2 min), (3) the H3 peak (∼22.1 min), and (4) the epimeric H4 double peak (∼22.6–22.8 min).

Quantification of the CLP metabolites H3 and H4 - contained in the supernatants from the metabolism of 2-oxo CLP with HLM - showed concentrations of the H3 metabolite of c_H3_ = 6.4 ± 0.2 µM and the H4 metabolite of c_H4_ = 8.6 ± 0.3 µM (first pre-study; *n* = 6). The ratio of the two detected H-metabolites was 42.7 ± 0.4% and 57.3 ± 0.4% for H3 and H4, respectively. On average, 66.8 ± 6.3 µM residual 2-oxo CLP (sum of both diastereomers) was detected in the supernatants after HLM metabolization. The quantification of the respective metabolites from each sample is shown in Figure 1A (HLM_1 – HLM_6). In the cis-CLP MP spiked reference sample used for the identification of the H-metabolites, only H3 and H4 were detected, but no traces of CLP, 2-oxo CLP or other metabolites. The signal intensity of the internal standard phenacetin was constant with a mean peak area of 274,331 ± 6,271 between the samples. The maximum deviation was detected at HLM_1 with 4.02%.

In the MPB-derivatized chromatograms of the HLM supernatants, 2-oxo CLP was detected as a diastereomeric double peak at 19.4 and 19.9 min, H3 as a single peak at 22.1 min, and H4 as a closely spaced double peak at 22.6 and 22.8 min (Figure 1). The occurrence of a double peak exclusively at the H4 position, but not for H3 or 2-oxo CLP, supports a substance-specific phenomenon rather than unspecific HPLC artefacts (e.g., partial column blockage or overloading, which would be expected to affect multiple peaks in the same run). This observation is consistent with the known stereochemical complexity of the active CLP thiol metabolite family: Pereillo et al. demonstrated that the active metabolite belongs to a family of eight stereoisomers generated from 2-oxo CLP, of which only one (bearing 7S, 3Z and a defined configuration at C4) retains biological activity (H4), while H3 shares 7S and 3Z configuration but differs at C4 and is inactive (29). Likewise, Zhang et al. described several chromatographically distinct active metabolite forms (M2–M5) with identical m/z 356 consistent with multiple diastereomers of the active thiol metabolite (39). In line with these findings, we interpret the H4 double peak in our assay as two closely related stereochemical forms (epimeric MPB derivatives and/or diastereomers at C4) of the same H4 stereoisomer, generated during bioactivation and derivatization. For quantification, the areas of both H4-related peaks were therefore summed. Two additional CLP-related signals were observed at 20.7 and 21.2 min. these peaks were absent in the cis-CLP MP reference and are therefore considered to represent additional, as yet unidentified MPB-derivatized CLP metabolites formed during the HLM incubation. Despite their presence, the HLM metabolization protocol yielded highly reproducible H3 and H4 concentrations and a stable H3/H4 ratio across six independent preparations, which we defined as a reproducible and sufficient yield for subsequent functional platelet aggregation experiments.

### 3.2 Characterization of blood specimens (pre-analytics)

Before a subject was included in the study, it was ensured that the platelet function was within the reference range (second pre-study). The PFA-100® was used to check platelet function immediately before the start of the experiments. The PFA-100® system measures the complex process of primary hemostasis and platelet dysfunction. The collagen + ADP induced platelet adhesion/aggregation time leading to occlusion of an artificial capillary was 86.2 ± 18.5 s (reference value for apparently healthy subjects: <120 s, the collagen + epinephrine induced occlusion time was 117.7 ± 28.3 s (reference range for apparently healthy subjects: <120 s) [23]. Both values confirmed normal platelet function. Also, the maximum of the platelet aggregation (TA_max_) with 93.2 ± 9.5% as well as the slope of the aggregation curve with 124.9 ± 19.8 degree/min were in the reference range for every single participant.

### 3.3 Influence of KPO_4_ buffer on platelet aggregation

As the H4 metabolite was dissolved in KPO_4_ buffer, another pre-study was carried out to test whether the addition of KPO_4_ to PRP affected platelet function (third pre-study). For this purpose, KPO_4_ (0.1 mM, pH 7.4) was added to the PRP of *n* = 3 apparently healthy subjects at 6 different volume fractions of 1, 2, 5, 10, 20, 50% (v/v) and the two parameters (TA_max_ and STA) were quantified (Table 1).

**Table 1:** Effect of potassium phosphate buffer (KPO_4_) concentration in PRP on ADP-induced platelet aggregation in *n* = 3 apparently healthy subjects (significance tests refer to the 0% (v/v) KPO_4_ value).

KPO_4_	TA_max_		STA	
[% (v/v)]	[%]	*p* =	[degree/min]	*p* =
0	92.4 ± 6.8		101.4 ± 23.5	
1	91.4 ± 7.3	0.86	101.8 ± 22.1	0.98
2	93.2 ± 8.8	0.91	101.9 ± 26.1	0.98
5	94.6 ± 1.7	0.62	99.7 ± 22.7	0.93
10	95.4 ± 3.4	0.54	107.9 ± 28.4	0.77
20	98.9 ± 5.4	0.26	118.6 ± 32.5	0.49
50	99.7 ± 1.3	0.14	120.2 ± 29.7	0.44

The results showed that up to a concentration of 50% (v/v) there was no significant effect on platelet aggregation (t-test in each case compared to control).

### 3.4 Influence of the vehicle on platelet aggregation

A similar investigation was carried out in the fourth pre-study for the addition of KPO_4_ + all additives of the HLM-metabolization, except the substrate 2-oxo-clopidogrel, to PRP (Table 2). Again, there was no effect of the added substances compared to the original medium.

**Table 2:** The impact of potassium phosphate buffer (KPO_4_) concentration, together with all additives of the human liver microsomes -metabolization, except the substrate 2-oxo-clopidogrel, on ADP-induced platelet aggregation in PRP of *n* = 3 apparently healthy subjects (significance tests refer to the 0% (v/v) KPO_4_ value).

KPO_4_ + additives	TA_max_		STA	
[% (v/v)]	[%]	*p* =	[degree/min]	*p* =
0	91.8 ± 9.9		110.2 ± 14.5	
1	87.2 ± 2.7	0.48	96.4 ± 13.9	0.3
2	92.0 ± 3.6	0.97	98.8 ± 14.4	0.39
5	96.2 ± 0.67	0.48	105.9 ± 15.4	0.75
10	96.9 ± 4.8	0.46	110.2 ± 13.9	0.99
20	92.1 ± 11.8	0.98	112.5 ± 9.0	0.83
50	91.2 ± 3.7	0.92	108.5 ± 21.8	0.57

### 3.5 Effective concentration of H4 on human platelet aggregation

In a further test, the effective concentration of the active H4 metabolite of CLP to inhibit ADP-induced platelet aggregation was determined (fifth pre-study; *n* = 3 apparently healthy subjects). For this purpose, PRP was preincubated with the active HLM-produced H4 metabolite. Five minutes later, ADP was added to activate the platelets and induce aggregation.

Two concentrations were chosen for this purpose: 2.15 µM and 4.3 µM which corresponds to 25% (v/v) and 50% (v/v) of the HLM-supernatant in PRP. Already at the concentration of 2.15 µM, the maximum platelet aggregation TA_max_ decreased from 93.2 ± 9.5% to 81.3 ± 2.4% (*p* = 0.092). At the concentration of 4.3 µM, TA_max_ decreased further from 93.3 ± 9.0% to 41.9 ± 22.6% (*p* = 8.8 × 10^−18^). Therefore, this concentration was used for the following study.

**Figure 2:**
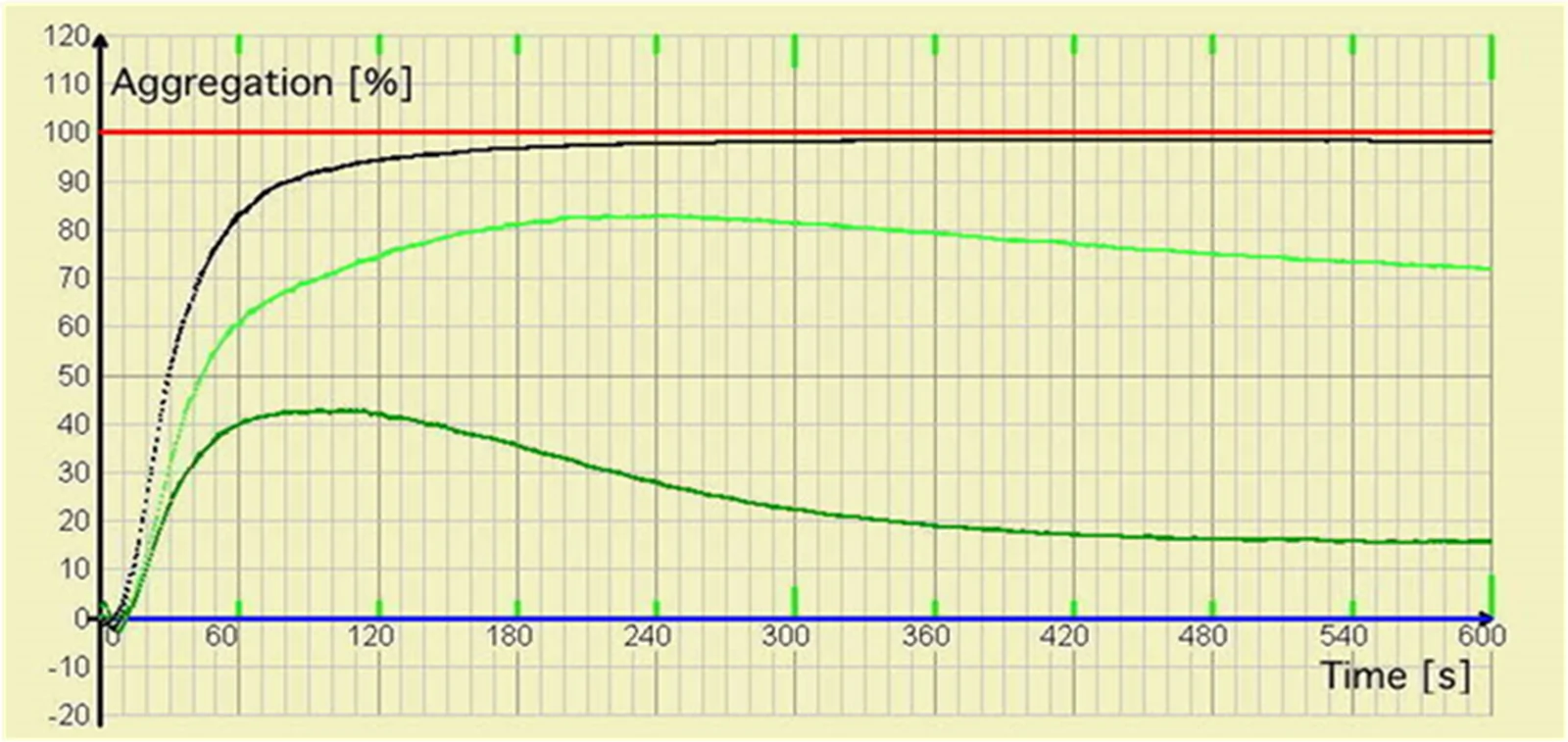
Representative adenosinediphosphate (ADP) - induced platelet aggregation (20 µM ADP) after 5 min incubation with the HLM-generated active H4 metabolite of CLP in two concentrations (2.15 µM in light green; 4.3 µM in dark green) compared to the vehicle treated ADP-induced platelet aggregation (black curve).

Figure 2 shows typical curves of ADP-induced platelet aggregation after treatment of PRP with both H4 metabolite concentrations in comparison to vehicle treated ADP-induced platelet aggregation.

### 3.6 Confirmatory, placebo-controlled *ex vivo* parallel group study

The confirmatory study was performed in accordance with the established and approved protocol. Table 3 shows the confirmatory parameter TA_max_ and the explorative parameter STA both after addition of ADP (20 µM) to PRP with or without the active H4 metabolite (4.3 µM). Supplementation of the active H4 metabolite to the PRP reduced TA_max_ after induction with ADP by an average of 55% (*p* = 8.81 × 10^−18^) and the slope of the aggregation curve STA by 35.5 degree/min (*p* = 6.86 × 10^−9^) compared to the vehicle. The standardized difference according to Cohen showed that the influence of the H4 metabolite on the ADP-induced platelet aggregation was highly relevant with 5.4 for TA_max_ and for STA with 2.23.

**Table 3:** ADP-induced (20 µM) platelet aggregation in PRP after 5 min incubation without (vehicle) or with addition of 4.3 µM of the active H4 metabolite of CLP (*n* = 8 subjects with 2 independent measurements each).

		Platelets + vehicle without active metabolite (H4) + ADP (20 µM)	Platelets + vehicle with active metabolite (H4) + ADP (20 µM)	*p* =
TA_max_	[%]	93.2 ± 9.5	41.9 ± 22.6	8.81 × 10^−18^
STA	[degree/min]	124.9 ± 19.8	80.6 ± 32.3	6.86 × 10^−9^

## 4 Discussion

Clopidogrel is an irreversible inhibitor of the platelet P2Y_12_ adenosine diphosphate receptor and is broadly used clinically for the prevention of thrombotic events in high-risk patients (40). The pharmacological target of clopidogrel is the ADP-induced platelet activation pathway. By selectively and irreversibly blocking the P2Y_12_ receptor, clopidogrel inhibits the ADP-mediated activation of the GPIIb/IIIa complex by preventing the binding of ADP to the P2Y_12_ receptor and thus blocking the binding site for fibrinogen (15). However, clopidogrel is a prodrug that requires bioactivation by cytochrome P450 (CYP)-enzymes to a pharmacologically active metabolite, the H4 metabolite (13). It is this metabolite that blocks the P2Y_12_ platelet receptor and thus prevents platelet aggregation. In patients with a certain genetic *CYP2C19* polymorphism, leading to reduced enzyme activity, the levels of the active H4-metabolite are significantly decreased, so that the inhibition of platelet aggregation is markedly reduced and a higher rate of adverse cardiovascular events may occur, indicating reduced clinical efficacy (8, 13, 41). This is supported by data from acute ischemic stroke patients, in whom CYP2C19*2/*3 carrier status was identified as risk factor for clopidogrel resistance (17). Importantly, a randomized controlled trial in patients with ischemic stroke or transient ischemic attack (TIA) showed that pharmacogenomics-guided antiplatelet therapy based on *CYP2C19* genotyping and clinical characteristics can significantly reduce recurrent stroke and composite vascular events and lower disability rates, without increasing bleeding complications, compared with standard treatment (18).

In this confirmatory, placebo-controlled *ex vivo* study, we established a HLM *in vitro* system capable of producing sufficient H4 metabolites to investigate the extent to which H4 inhibits platelet aggregation and the kinetics of this inhibition. Importantly, the H4 metabolite was generated using a standardized protocol with a single, well-characterized batch of pooled HLM and common single-use aliquots of all critical reagents, which resulted in highly reproducible H3 and H4 yields in independent metabolization reactions and thereby supports the reliability of the experimental approach. The extent of the inhibition is comparable to the effect of 600 mg clopidogrel as a loading dose on ADP-induced platelet aggregation measured in coronary patients (5).

The concentration of the H4 metabolite of 4.3 µM – a concentration that was found to effectively inhibit platelet aggregation in an earlier study (15) - corresponds to a concentration of 1,530 ng/mL in the PRP, as the supernatants of the human liver microsomes were diluted 1:1 with the platelets in the mixture. This is much higher than the 171 (116–215) ng/mL H4 metabolite detected in patient´s plasma 60 min after a loading dose of 600 mg Clopidogrel. Ten hours later neither clopidogrel nor its active metabolite could be detected in plasma of healthy volunteers (42). This difference is probably mainly since only the unbound H4 metabolites are measured in blood plasma. As H4 metabolites are comparatively hydrophobic, most of them will be bound to plasma proteins (approximately 98%), platelets, erythrocytes or endothelial cells and thus escape quantification (43). In the present study, however, the concentration of H4 metabolites was measured in a largely protein-free solution without cells.

Clopidogrel action depends on dose and time. Repeated daily doses of 75 mg will take about 7 days for maximum platelet inhibition (44, 45). Pareek et al. were able to show that the inhibition of ADP-induced platelet aggregation was 18.5% after 6 h and 51.1% on day 3 (at a loading dose of 300 mg/d and a maintenance dose of 75 mg/d) (46). This delayed onset of the effect on platelet aggregation after oral administration of clopidogrel (47) can be considerably reduced to minutes by direct administration of the H4 metabolite (see Figure 2 and Table 3). Five minutes after incubation of PRP with HLM-derived H4 metabolite and ADP activation, an inhibition of platelet aggregation >55% occurred. *In vivo* in responders to 600 mg clopidogrel, this was not seen until 6 h later with a comparable potency (46). This could eliminate the need for premature administration of clopidogrel prior to an intervention and thus greatly simplify the patient preparation of the cardiac catheterization laboratory. Direct administration of H4 might therefore be particularly advantageous in patients at high risk for clopidogrel resistance, such as carriers of CYP2C19*2/*3 alleles, as recently demonstrated in acute ischemic stroke cohorts (17). While genotype-guided adjustment of oral antiplatelet therapy has already been shown to improve outcomes in stroke and TIA patients (18), an intravenous administration of the active H4 metabolite could offer an alternative, genotype-independent strategy to rapidly achieve effective platelet inhibition, particularly in acute settings or when timely genotyping is not feasible.

The H4-dependent changes in platelet aggregation measured in the present study appeared to be mostly attributable to the effect of the H4 metabolite and were recorded within the scope of the measurement accuracy. The vehicle solution of the H4 metabolite (see Tables 1, 2) was found to have no significant impact on the results. A significant influence of remaining substrate 2-oxo-clopidogrel can also be excluded, since treatment with 250 µM showed no influence on platelet function. Furthermore, the influence of platelet ageing on the results can be excluded, since all experiments were conducted during a four-hour time-window after the blood donation. Despite ageing of platelets is a continuous process in blood samples, this time-frame is considered adequate for reproducible platelet function test (35). Furthermore, platelet functionality was always controlled at the end of a series of experiments.

From a structural perspective, our HPLC findings are in agreement with previous work on the active clopidogrel thiol metabolite family. Pereillo et al. showed that bioactivation of 2-oxo-clopidogrel by human liver microsomes generates a set of eight stereoisomeric H-metabolites, of which only one (H4; 7S, 3Z, specific C4 configuration) is biologically active, whereas H3, despite sharing 7S and 3Z configuration, is inactive (29). Zhang et al. likewise reported multiple chromatographically distinct active metabolite forms (M2–M5; m/z 356) attributable to diastereomers of the same active thiol metabolite (39). In our study, this stereochemical heterogeneity is reflected by the selective appearance of H4 as a double peak, whereas H3 elute as single symmetrical peak and 2-oxo CLP as two diastereomeric peaks. This pattern supports a stereochemical explanation (two closely related stereoisomeric/epimeric forms of H4) rather than an unspecific chromatographic artefact and is compatible with treating the summed H4 peak area as one analytically and pharmacologically relevant entity.

Consistent with these functional data, HPLC analysis demonstrated reproducible formation of H4 (together with the inactive diastereomer H3) as the predominant thiol-containing metabolite in the HLM supernatants. While additional clopidogrel-related metabolites with intermediate retention times were detected, their chemical structures and pharmacological properties remain to be clarified. The absence of any effect of the vehicle or 2-oxo-clopidogrel alone, the known lack of antiplatelet activity of H3, and the tight correlation between the reproducible H4 concentrations and the observed inhibition of ADP-induced platelet aggregation collectively support the conclusion that H4 is the main driver of the antiplatelet effect, even though a contributory role of co-formed metabolites cannot be completely excluded.

An important aspect of the present *in vitro* approach is the chemical lability of the H4 metabolite. To minimize potential degradation, H4-containing HLM supernatants were always generated freshly on the day of experimentation, stored on ice at 4 °C for no longer than 4 h, and used immediately for platelet aggregation assays. In addition, thiol-containing metabolites were derivatized directly after metabolization for HPLC analysis to avoid artifactual loss during analytical processing. Under these strictly controlled conditions, we observed highly consistent H4 concentrations in the HPLC analyses and reproducible inhibitory effects on ADP-induced platelet aggregation across donors, indicating sufficient functional stability of the metabolite within the time frame of our experiments.

Limitations of the study:

As this is an *ex vivo* study, it is not possible to extrapolate the results to *in vivo* conditions. All subjects included in the study were apparently healthy subjects so that possibly different results may occur in platelets from patients with cardiovascular or hematological diseases. Finally, the sample size of the study was small. Therefore, the study should be considered as proof-of-concept study with all its conclusions requiring further external validation. In addition, although we used a single commercially characterized batch of pooled HLM and observed highly reproducible H3/H4 formation, we did not perform a separate systematic inter-lot comparison of microsomal preparations or a dedicated stability study of H4 under varying experimental conditions. Moreover, the additional clopidogrel-related metabolites detected in the HPLC chromatograms were not structurally elucidated, and their potential pharmacological contribution cannot be fully ruled out. More comprehensive characterization of microsomal variability, H4 stability, and co-formed metabolites should therefore be addressed in future studies, particularly in the context of methodological standardization and clinical translation.

## 5 Conclusion

Early and relevant platelet P2Y_12_ receptor inhibition is an effective approach to avoid thrombus formation. The active H4 metabolite of clopidogrel – which was metabolized by human liver microsomes from 2-oxo-clopidogrel – resulted in significant and relevant inhibition of ADP-induced platelet aggregation *in vitro* with a very short latency period. With the direct administration of the H4 metabolite, the limitations of clopidogrel – the high interindividual variability due to genetic polymorphisms of *CYP2C19*, delayed onset of action, and inadequate efficiency (5, 40, 48) – could be largely avoided.

## Data Availability

The raw data supporting the conclusions of this article will be made available by the authors, without undue reservation.
